# Public Health Awards for the Asia-Pacific Region From the World Health Organization

**DOI:** 10.1177/10105395231184676

**Published:** 2023-07-05

**Authors:** Colin Binns, Wah Yun Low

**Affiliations:** 1 School of Public Health, Curtin University, Perth, WA, Australia; 2 Faculty of Medicine, University of Malaya, Kuala Lumpur, Malaysia

Each year at the Annual Assembly of the World Health Organization (WHO), the winners of public health awards are announced. These awards recognize people and institutions who have made significant contributions to global public health.

Dr Tedros Adhanom Ghebreyesus, WHO Director-General, when announcing the awards in May 2023 stated, “At a time when the world faces many challenges, each is an inspiration and a reminder of the progress that can be made to improve health and wellbeing for all.”

This year, there are two individuals and two institutional awards from the Asia-Pacific region. Professor Dr Vichai Tienthavorn is the President of the Praboromarajchanok Institute at the Ministry of Health Thailand; which undertakes research and education in health problems of importance to Thailand. He has worked on nation-wide health-promotion initiatives to address thalassemia, diabetes, and hypertension. Dr Maria Asuncion Silvestre from the Philippines was awarded the United Arab Emirates Health Foundation Prize for her work in promoting breastfeeding.

Under the leadership of Professor Dr Vichai Tienthavorn, the Praboromarajchanok Institute has focussed on the changing health issues of Thailand from communicable to noncommunicable diseases.^1^ His recognition focuses on two directions of his work, the efficient and effective management of a genetic disease program (thalassemia) and several lifestyle modification programs for diabetes.

Thalassemia is a monogenetic disorder caused by mutation of a single gene. Another common disorder which is also monogenic is familial hypercholesterolemia. The name thalassemia derives from its first descriptions around the Mediterranean Sea, being a combination of Greek words for blood and water (sea). Clinical thalassemia since then have been shown to stretch in a band across the Southern Mediterranean as far west as the Philippines (WHO map).^2^ Thailand has a population of 66.2 million with 30.0% to 40.0% of them carrying thalassemia genes, creating the need for a major public health program.

Thalassemia results from the gene interactions to cause a wide spectrum of clinical severity from asymptomatic to lethal. It is estimated that about 1.2% of Thai babies are born with severe cases of thalassemia each year. The overall population prevalence of thalassemia disease is about 1.0%. The etiology of diabetes is more complex and involves the interaction of complex genetics and lifestyle factors.

Thalassemia prevention and control programs in Thailand were introduced using postconception screening in couples and prenatal diagnosis for the prevention of new thalassemic births.^4^ The management of existing cases is currently based on supportive treatment with regular blood transfusions and iron chelation. This involves the coordination of many facilities across the country. Curative treatment by hematopoietic stem cell transplantation is possible but is not yet universally available.^5^

Thalassemia is also a major health problem in other Asia-Pacific countries. Malaysia is a multiethnic nation with a population of 34 million, and it is estimated that 6.8% of its population carry thalassemia genes. There are 8 000 registered patients, with the largest concentration in the state of Sabah.^6^ The Philippines also has a high prevalence of thalassemia gene carriers and has a national newborn screening program. However, the large population (110 million) is scattered across nearly 8 000 islands, making the implementation of a national blood transfusion strategy very difficult.^7^

Diabetes is an increasing health problem across our region. The *Asia Pacific Journal of Public Health* has published more than 200 articles documenting its prevalence and providing information on public health programs. In 2021, Thailand was estimated to have 6.1 million people with diabetes (age 20-79 years); up from 4.1 million in 2011.^3^ The etiology of diabetes involves the interaction of complex genetics and lifestyle factors. Health promotion programs provide best ways of reducing the population burden of thalassaemia and diabetes. It is fitting that the work of one of the pioneers of health promotion programs, Professor Dr Vichai Tienthavorn, has been recognized by the WHO.

The following table shows the rapid increase in diabetes in our region, where some of the island countries have the highest rates in the world (Table 1).^8^

**Table 1.** Prevalence of Diabetes in Asian Countries.

Diabetes (20-79 years)	2011	2021
China	90m	140.9m
Indonesia	7.3m	19.5m
Japan	10.7m	11m
Thailand	4m	6.1m
Malaysia	2m	4.4m

Source: Data from International Diabetes Federation Atlas, 2021.^3^

Abbreviation: m, million.

Dr Maria Asuncion Silvestre is the founder and remains the President of “Kalusugan ng Mag-Ina Inc.” (Health of Mother and Child), a nongovernmental organization devoted to promoting exclusive breastfeeding and appropriate neonatal care. The *Asia Pacific Journal of Public Health* has published several articles on the benefits of exclusive breastfeeding from birth, to reduce early childhood morbidity and mortality and to contribute to a healthy life. Exclusive breastfeeding also contributes to the health of mothers. Dr Maria Asuncion Silvestre has devoted her life to promoting exclusive breastfeeding and early essential newborn care.

The works of both awardees are related across the broad spectrum of public health needs. As many articles in this journal have shown, universal adoption of WHO breastfeeding guidelines will significantly reduce the population burden of chronic diseases, including diabetes.

We congratulate Professor Dr Vichai Tienthavorn and Dr Maria Asuncion Silvestre on their awards. They both reflect the broad spectrum of public health and the need to actively promoting public health practices.

This issue of the journal includes more articles on the ravages that smoking reaps on our communities, both on those who smoke and those who are forced to breathe air that is contaminated by those who smoke. In addition, there is an article on COVID-19. The WHO states that cases of COVID-19 are continuing to be reported all around the world.^9^ We anticipate that there will be another peak in the Southern Hemisphere during the winter, combined with influenza and again in the Northern Hemisphere later in the year. Immunity from the 13-billion doses of vaccine administered so far has started to wear off, and in particular, the elderlies are proving to be vulnerable to infection again. We expect that this journal will continue to publish COVID-19 articles for some time into the future.
